# Epidermal growth factor receptors in intracranial and breast tumours: their clinical significance.

**DOI:** 10.1038/bjc.1991.130

**Published:** 1991-04

**Authors:** R. A. Hawkins, E. Killen, I. R. Whittle, W. J. Jack, U. Chetty, R. J. Prescott

**Affiliations:** University Department of Surgery, Royal Infirmary of Edinburgh, UK.

## Abstract

A method to determine the binding of epidermal growth factor (EGF) to the particulate fraction of the cell has been established and evaluated using rat liver, human placenta, and tumours of human breast and brain. Little EGF receptor (EGFR) activity was detected in normal or benign tumour tissues except for meningioma (positive in 95% samples), but EGFR were present in 43% of 131 breast tumours and 75% of 55 primary cerebral tumours. Despite the strong inverse correlation between EGFR activity and oestrogen receptors in breast tumours and a tendency for high levels of EGFR activity to be associated with glioblastoma multiforme, analysis showed that EGFR was of little prognostic significance in patients with tumours of either breast or brain.


Br. J. Cancer (1991), 63, 553-560 © Macmillan Press Ltd., 1991

### Epidermal growth factor receptors in intracranial and breast tumours: their clinical significance

R.A. Hawkins[1], E. Killen[1], I.R. Whittle[2], W.J.L. Jack[3], U. Chetty[1] & R.J. Prescott[4]

[1]*University Department of Surgery, Royal Infirmary of Edinburgh;* [2]*Department of Clinical Neurology, Western General Hospital, Edinburgh;* [3]*Scottish Cancer Trials Office (MRC), Edinburgh;* [4]*Medical Statistics Unit, University of Edinburgh, Edinburgh, UK.*

**Summary** A method to determine the binding of epidermal growth factor (EGF) to the particulate fraction of the cell has been established and evaluated using rat liver, human placenta, and tumours of human breast and brain. Little EGF receptor (EGFR) activity was detected in normal or benign tumour tissues except for meningioma (positive in 95% samples), but EGFR were present in 43% of 131 breast tumours and 75% of 55 primary cerebral tumours. Despite the strong inverse correlation between EGFR activity and oestrogen receptors in breast tumours and a tendency for high levels of EGFR activity to be associated with glioblastoma multiforme, analysis showed that EGFR was of little prognostic significance in patients with tumours of either breast or brain.

Recent advances in our understanding of the ways in which alterations in DNA can lead to cancer have suggested two major mechanisms (Ponder, 1988) namely (1) the overactivity of certain genes ('oncogenes') involved in transducing the signals for growth and (2) the loss of other genes ('tumour suppressor' or 'anti-oncogenes') which regulate differentiation and limit growth. One putative component of the former mechanism is epidermal growth factor (EGF), a small ($M_r$ 6,045) peptide which acts through a larger, cell-surface receptor protein (EGFR, $M_r$ 180,000). This receptor protein is structurally related to the product of a known oncogene (v-*erb* $B_1$). Potentially, overexpression of either the growth factor or its receptor could contribute to the process of carcinogenesis. Reports in the literature have suggested that over-expression of the receptor protein may occur in several types of tumour (Neal *et al.*, 1985; Hwang *et al.*, 1986; Cowley *et al.*, 1986), including those of the brain (Lieberman *et al.*, 1985) and breast (Sainsbury *et al.*, 1985). Whether the growth factor or its receptor are involved in carcinogenesis in man is as yet unclear.

In established breast tumours, expression of the EGF receptor protein has been reported to be an index of poor prognosis (Sainsbury *et al.*, 1985; 1987), likelihood of lymph node involvement (Battaglia *et al.*, 1988) and lack of response to endocrine therapy (Nicholson *et al.*, 1989). The expression of EGF receptors shows a strong inverse correlation with that of oestrogen receptors (Sainsbury *et al.*, 1985; Perez *et al.*, 1984; Delarue *et al.*, 1988) and thus to some extent, measurements of EGFR may provide the same information as that derived from ER measurements.

In malignant gliomata, over-expression of the EGFR protein appears to be associated with gene amplification (Wong *et al.*, 1987). In culture, the growth of glial tumour cells can be stimulated by EGF (Frappaz *et al.*, 1988) and antibodies against the EGFR can inhibit such stimulation (Werner *et al.*, 1988). Clinically, an antibody against the EGFR, labelled with $^{131}I$, has been reported to have induced significant regression of a glioma in a young man (Epenetos *et al.*, 1985), though the specificity of this effect is uncertain.

In view of the above considerations, we set out (1) to measure the levels of EGFR activity in series of intracranial and breast tumours and (2) to relate these findings to the patient's outcome. In view of the established significance of the oestrogen receptor (Knight *et al.*, 1977; Adami *et al.*, 1985), we have also examined the results for this protein in the breast tumours.

Correspondence: R.A. Hawkins.
Received 9 July 1990; and in revised form 12 November 1990.

### Materials and methods

### Radiochemicals

Initially, $^{125}I$ iodide, carrier-free (Cat. No IMS 30) was purchased from Amersham International, Little Chalfont, Bucks. and used to prepare $^{125}I$ EGF. A total of 19 iodinations were carried out using either iodogen (Fraker & Speck, 1978; $n = 16$) or lactoperoxidase (Thorell & Johnson, 1971; $n = 3$). The radioactive EGF thus prepared was stored in aliquots at $-40°C$ until required, for up to 1 month. The specific radioactivity of the preparations used ranged from 76 to 196 $\mu Ci\ \mu g^{-1}$ i.e. 2.8–7.3 $MBq\ \mu g^{-1}$. Because of the unpredictability of these iodinations in our hands, we changed over to purchasing the radioligand.

$^{125}I$ EGF (152–177 $\mu Ci\ \mu g^{-1}$ i.e. 5.6–6.6 $MBq\ \mu g^{-1}$, Cat. No NEX-160) was purchased from Du Pont (UK) Ltd, Wedgewood Way, Stevenage, Herts. and used without repurification.

[2,4,6,7-$^3H$]oestradiol-17$\beta$ (85–110 $Ci\ mmol^{-1}$ or 3.1–4.1 TBlq mmol, Cat. No TRK 322) was purchased from Amersham International and repurified on Sephadex LH-20 (Mikhail *et al.*, 1971) at 3-weekly intervals.

### Non-radioactive chemicals

Epidermal growth factor was obtained in three forms: (1) mouse EGF, tissue culture grade (Cat. No E7755) and (2) mouse EGF, receptor grade (Cat. No E6135) were both obtained from the Sigma Chemical Co. Poole, Dorset, UK, while (3) human EGF ($M_r$ 6216) was a recombinant preparation given by Dr H. Gregory of ICI Ltd, Pharmaceuticals Division, Macclesfield, Cheshire.

Iodogen (1,3,4,6 tetrachloro-3α-6α-diphenyl glycouril) was obtained from the Sigma Chemical Company.

Sephadex G-25 and LH-20 were obtained from Pharmacia Ltd, Milton Keynes, Bucks.

All other chemicals were obtained from either the Sigma Chemical Company or from BDH Ltd, Poole, Dorset.

### Patients

*(a) With intracranial tumours* Tissue specimens of intracranial neoplasms were obtained from 88 patients undergoing surgery in the Department of Clinical Neurology, Western General Hospital, Edinburgh. A total of 96 specimens were obtained. The patients ranged from 2 to 84 years of age and the specimens were classified according to Burger *et al.* (1985) and Russell and Rubenstein (1989).

In 11 patients, specimens of normal, peri-tumoral brain were available from the material excised at operation (eight specimens from patients where tumour was also available and three where no other tissue was available). The remaining 85 abnormal tissues included 51 gliomata which were further divided into 47 commoner types and four rarer types.

Follow-up time for all these patients ranged from 1–105 months (median 9 months). Follow-up was analysed only for the 51 patients with tumours of the commoner types, i.e. 47 commoner gliomata and four primitive neuro-ectodermal tumours (PNETs), since the natural history of meningiomas and schwannomas necessitates long-term follow-up to document recurrence (Simpson, 1957). In these patients, surgery was by craniotomy and debulking in all patients except one with a PNET who had craniotomy and biopsy only.

*(b) With tumours of the breast* For 151 female patients undergoing surgery for a lesion of the breast between 16-09-86 and 24-06-88, a specimen was submitted for oestrogen receptor analysis and sufficient material was present for the additional assay of EGFR. Of these patients, nine had benign lesions, one had a melanoma, five had specimens containing an inadequate sample (<10%) of tumour (Steele *et al.*, 1987), two had squamous carcinoma, one had a malignant histiocytoma, one bilateral breast carcinoma and for one patient, no notes were available.

For the remaining 131 cases with a single breast cancer and adequate tumour specimen, tumour stage ranged from $T_{1s}$ to $T_4$ and age from 27 to 87 years. One case had been treated initially in 1959 and was also omitted from analysis. Some data were missing in a further four cases and six had metastases ($M_1$) at presentation. The net result was that for the study of survival, 126 cases were available and for that of disease-free interval 123 (life-table analysis) or 120 (univariate and multivariate analysis).

Follow-up time for this mixed group of patients was relatively short (median 20, range 2–73 months).

### *Determination of EGF receptor activity*

The method used is based on that of Sainsbury *et al.* (1985). After examination of the steps involved in the assay separately, the following procedure was adopted routinely.

*(1) Preparation of 'membranes'* In general, 200 to 600 mg of fat-free tissue were chopped and homogenised in 2 ml tris-monothioglycerol-glycerol buffer (tris 10 mM, 0.25 M sucrose, 1 mM EDTA, pH 8.0, plus 1% v/v monothioglycerol and 10% v/v glycerol) using one burst of 20 s, 1 min pause for cooling and a second burst of 15 s, maximum speed in a Silverson metal homogeniser. The homogenate was kept on ice and filtered through a coarse metal sieve to remove lumps. The homogeniser and sieve were washed in 2 × 1.0 ml of buffer and the homogenate plus washings was remixed and centrifuged for 30 min at 105,000 *g* and 4°C in a Beckman TL100 ultracentrifuge.

The resulting pellet ('membranes' = total particulate fraction) was resuspended in tris-saline buffer (10 mM, 0.15 M NaCl, pH 7.4) to give a final concentration of 200–266 mg tissue $ml^{-1}$ for breast tissues (in seven tissues only 140–190 mg $ml^{-1}$ was possible) or 50–250 mg tissue $ml^{-1}$ for brain tissues. The resuspended 'membranes' were rehomogenised gently by hand in a glass-glass homogeniser to ensure complete resuspension.

*(2) Incubation* One hundred microlitre portions of 'membranes' (i.e. generally = 20–26 mg tissue per tube for breast tissues and = 5–25 mg tissue per tube for brain tissues) were distributed, where possible, into each of 15 tubes, containing 200 µl non-radioactive EGF solution (to yield final concentrations of 0.024, 0.049, 0.098, 0.195, 0.39, 0.78, 1.56, 3.125, 6.25, 12.5, 25.0, 50.0, 100.0 or 300 nMolar), and 100 µl of $^{125}I$ EGF (approximately 10,000 c.p.m., to yield a final concentration of approximately 0.02 nMolar), on ice. The contents of each tube (total volume 400 µl) were mixed and the tubes were incubated for 90 min at 26°C.

*(3) Termination* All tubes were placed on ice and to each was added 0.5 ml of immunoglobulin G solution (0.5% w/v) and 1.0 ml of polyethylene glycol solution (25% v/v) with mixing between the additions. The tubes were remixed and centrifuged immediately for 15 min at 2,100 *g* in the swing-out-rotor of a precooled, MSE Mistral 6L centrifuge. After centrifugation, the supernatant (free) fraction was aspirated from each tube and the remaining pellet (bound) was counted for 2 × 10 min in a Packard Multi-Prias Gamma Counter at approximately 70% efficiency. Three tubes containing only $^{125}I$ EGF ('radioligand') were also counted to yield the starting c.p.m./tube.

*(4) Calculation of receptor site concentration* After correction of the counts bound in each tube by subtraction of the value for non-specific binding (300 nMolar tube), the data were subjected to Scatchard (1949) analysis. Non-specific binding averaged 8.9% of the total radioligand added ($n = 30$). EGFR activity was designated as positive when ⩾150 c.p.m. were displaced over at least four of 15 tubes by non-radioactive EGF in the initial (high affinity) part of the curve. Where no displacement was evident, the tissue was designated EGFR – negative. Cases showing only a small displacement and where a Scatchard plot was not possible were designated as 'EGFR equivocal'.

For quantitative purposes, routinely curves were drawn by hand and extrapolated to the horizontal axis to yield an estimate of the total binding sites (high + low affinity sites) present. Subsequently we examined many of the plots by the computer-analysis method of Hetherington (Sainsbury *et al.*, 1985; Nicholson *et al.*, 1988) to assign, fit and calculate both the slopes and x-axis intercepts where two components were present. Retrospective comparison of the extrapolation method with the computer – analysis method showed very little error (total sites from extrapolation = 98.1% value from computer analysis, $n = 17$ assays on rat liver membranes). 'EGFR – equivocal' tissues were arbitrarily assigned a total receptor site concentration of 5 fmol $mg^{-1}$ membrane protein, though in some EGFR – positive tissues, values as low as 1 fmol $mg^{-1}$ membrane protein were derived by calculation.

A sample of rat liver membranes was processed, as a quality control, with each batch of samples. These membranes, prepared in bulk and stored in aliquots at −40°C, lost activity gradually on storage (approx 47% over 10 months) but were nevertheless useful. The inter-assay coefficient of variation for total sites in rat liver (measured over only 1 month) was 21% ($n = 6$), 38% ($n = 7$), 23% ($n = 6$), and 30% ($n = 6$) for four different months, the mean loss of activity being 4.7% per month on storage.

In preliminary experiments, we investigated (a) the influence of the subcellular fraction chosen on binding, (b) three different methods for separating free and bound peptide, (c) the influence of time and temperature on binding and (d) the specificity of the binding observed.

### *Determination of oestrogen receptor activity*

Oestrogen receptor activity was determined by the method of Hawkins *et al.* (1975, modified 1981) on the supernatant remaining from EGFR assay or prepared by homogenisation of a separate, adjacent portion of tissue. The data were analysed according to Scatchard (1949) using a BBC microcomputer to yield the dissociation constant of binding (Kd nmols $l^{-1}$) and receptor site concentration (fmols $mg^{-1}$ soluble protein).,

Quality controls, consisting of pools of minced human myometrium were processed with every assay and the inter-assay coefficients of variation were 17.0% ($n = 48$) at high levels (111 fmol $mg^{-1}$ protein) and 25.5% ($n = 144$) at lower levels (48 fmol $mg^{-1}$ protein).

### *Determination of protein concentration*

The protein contents of membrane preparations were determined after dissolution in 2 N sodium hydroxide solution and neutralisation with 2 N HCl solution, by assay with Coomassie Blue reagent (Bradford, 1976) against a mixed protein standard (Sigma, Cat. No 540-10). Tissue extracts ('cytosols') were assayed directly. Five quality control samples were processed with each assay and where the mean value deviated by more than 10% from the expected, the assay was repeated.

*Statistical evaluations*

The inter-relationship between oestrogen and epidermal growth factor receptor activities was examined by Kendall's Rank Correlation Coefficient. The inter-relationship was also examined by classifying breast tumours as EGFR-positive ($\geqslant 1$ fmol mg$^{-1}$ protein) or EGFR-negative, and ER-positivity according to various cut-offs (5, 10, 15, 20, 30, 50 and 100 fmol mg$^{-1}$ protein). Cohen's kappa was then calculated (Cohen, 1960) to give a measure of the concordance between EGFR-positivity and ER-negativity.

In order to examine the importance of each receptor activity in relation to prognosis (disease-free interval and survival for breast tumours, survival for brain tumours), Cox's proportional hazards regression model was applied in firstly, univariate and secondly, multivariate analyses.

### Results

*Binding of $^{125}$I EGF in four types of tissue*

The binding of $^{125}$I EGF was determined in four kinds of tissue: rat liver and human placenta (controls) and tumours of breast and brain. Examples of the Scatchard plot obtained for each type of tissue are shown in Figure 1. Inspection of the plot revealed evidence for two binding compontents in some tissues (rat liver always, others more variable) and one in others. When the data were analysed by computer, the Kds of the high and low affinity components were in the ranges of 0.01–0.50 nM and 1.0–5.0 nM respectively (see Figure 2), though the affinity in some brain tumours was apparently rather different (see Discussion).

Since (1) initially we did not have the computer program for separating the two components (2) there is no conclusive evidence that lower affinity sites are of no significance, in the results which follow, concentration of total receptor sites has been calculated by extrapolation, unless stated otherwise.

*Influence of mode of separating free and bound ligand*

A comparison of three different methods for separating free and bound ligand was carried out on rat liver membranes. The methods were (A) simple centrifugation of the membranes (Sainsbury *et al.*, 1985) (B) filtration of the membranes (based on Fitzpatrick *et al.*, 1984) and (C) co-precipitation of IgG with polyethylene glycol (based on Carpenter, 1985). This showed that by method C, on average, slightly lower Kd values (stronger binding) for both low and high affinity components (ratios of $Kd_1$ by methods A: B: C = 3.49: 3.86: 1.00; $Kd_2$ = 1.82: 1.89: 1.00, means of four experiments by computer analysis) and higher numbers of total receptor sites (ratios A: B: C = 0.78: 0.87: 1.00) were observed than were found by methods A or B. The $Kd_1$ and $Kd_2$ values found by this method, C, were also more consistent and this method was selected for the routine procedure.

*Influence of subcellular fraction analysed*

When the total homogenate was separated by centrifugation into subcellular fractions, binding activity was found in all the particulate subcellular fractions (Table I). This was especially evident in rat liver and brain tumours of the three types of tissue examined. In view of this finding, for routine purposes, we collected all the particulate fractions ('membranes') of the cell for assay, by a single centrifugation at 108,000 *g*.

*Specificity of binding*

The specificity of binding of $^{125}$I EGF, tested in placenta, a breast tumour and three brain tumours (Figure 2), showed that there was no consistent increase in displacement of binding with increasing concentration of competing protein/peptide except with EGF among the compounds tested (Figure 2). Culture grade EGF, displaced 76–81% of the binding in placenta and a breast tumour and 29–44% in three brain tumours. In the latter tissues, displacement by both human and mouse EGF was in excess of 80% at 300 nM competitor for two of the three tumours examined, but only attained 55% in the third.

*Influence of temperature*

For three types of tissue, the time course of binding was examined at various temperatures (Figure 3a, b and c). Using a fixed, but non-saturating concentration of ligand (~0.15 nM), maximal specific binding was observed with rat liver 'membranes' after approximately 60 min at 26°C, lesser binding being observed at 4°C or 37°C (Figure 3a). In a breast tumour (Figure 3b), maximal binding was observed after approximately 40 min at either 26°C or 37°C and lower binding was observed at 4°C. For a brain tumour (Figure 3c), the

**Figure 1** Binding of $^{125}$I EGF to four types of tissue: Scatchard plots, analysed by computer for **a**, rat liver, **b**, placenta, **c**, breast tumour and **d**, brain tumour.

**Figure 2** Specificity of binding of $^{125}$I EGF to three types of tissue: **a**, placenta, **b**, breast tumour and **c**, brain tumour. Membranes from 5–36 mg tissue were exposed to 20,000 or 100,000 c.p.m. $^{121}$I EGF (0.05–1.0 nM) in the presence or absence of competing peptide (12.5–300 nM) for 90 min at 26°C.

time course of binding was different, binding being maximal (26°C or 37°C) or nearly maximal (4°C) after approximately 40 min and changing little thereafter. It was not possible to perform all of these studies at the outset, and thus the conditions of 90 min at 26°C, chosen for the routine assay, represent a compromise.

*Incidence and significance of EGFR in breast tissue*

Of nine samples of tissue designated 'normal/benign' on histopathological examination, only one (11%) contained a low level of EGFR activity.

Of the 131 samples from solitary breast cancers with adequate tumour content, 56 (43%) had EGFR activity (⩾1 fmol mg$^{-1}$ membrane protein) and 83 (63%) had ER activity (⩾5 fmol mg$^{-1}$ soluble protein). There was an inverse correlation between the presence of ER and that of EGFR (Kendall's R = −0.35, $P<0.001$). By Cohen's Kappa test, this inverse relationship was strongest (Cohen's Kappa 0.52–0.53) when a cut-off of 10–15 fmol mg$^{-1}$ protein was used to designate 'presence/absence' for the oestrogen receptor.

At analysis, there had been 43 recurrences and 30 deaths. When the relationship of the various clinical or biochemical parameters to disease-free interval or survival was examined by univariate analysis, only node status, tumour stage, tumour size and oestrogen receptor concentration had a significant influence (Table II). By multivariate analysis, only node status and oestrogen receptor concentration influenced prognosis (Table III). No clear relationship was shown between EGFR activity and prognosis whilst higher levels of ER activity (>100 fmol mg$^{-1}$ protein) were associated with a longer disease-free interval (survival curves not shown).

**Table I** Distribution of EGFR amongst the subcellular fractions in three types of tissue

| *Tissue type* | *Expt. no.* | *Total EGFR in all fractions (A)* | *% activity in each fraction* B | C | D | E |
|---|---|---|---|---|---|---|
| Rat liver | 1 | 18.1 | 40.7 | 33.2 | 26.1 | 0 |
| | 2 | 5.47 | 64.0 | 24.6 | 11.4 | 0 |
| | 3 | 16.2 | 52.8 | 35.9 | 11.3 | 0 |
| | 4 | 2.26 | 31.4 | 15.5 | 53.1 | 0 |
| | 5 | 7.35 | 24.9 | 42.8 | 30.5 | 1.8 |
| | 6 | 6.91 | 31.7 | 22.3 | 46.0 | 0 |
| Mean | | | 40.9 | 29.1 | 29.7 | 0.3 |
| Breast tumours | 1 | 16.8 | 5.0 | 5.1 | 89.9 | 0 |
| | 2 | 2.96 | 3.0 | 33.8 | 63.2 | 0 |
| | 3 | 2.97 | 33.6 | 12.5 | 53.9 | 0 |
| | 4 | 0.59 | 32.5 | 30.8 | 36.7 | 0 |
| Mean | | | 18.5 | 20.6 | 60.9 | 0 |
| Brain tumours | 1 | 94.6 | 29.0 | 33.5 | 31.7 | 5.8 |
| | 2 | 40.6 | 35.4 | 27.7 | 36.9 | 0 |
| | 3 | 1335.6 | 28.0 | 36.7 | 33.4 | 1.9 |
| | 4 | 24.3 | 30.0 | 39.0 | 31.0 | 0 |
| | 5 | 265.0 | 47.3 | 36.6 | 16.1 | 0 |
| Mean | | | 33.9 | 34.7 | 29.8 | 1.6 |

A = sum of total receptor sites derived by extrapolation of a Scatchard plot for each fraction, expressed as fmol sites mg$^{-1}$ wet tissue; B = 'nuclei/débris', 800 *g* pellet; C = 'low speed membranes', 800–2,000 *g* pellet; D = 'high speed membranes', 2,000–108,000 *g* pellet; E = 'cytosol', 108,000 *g* supernatant.

*Incidence and significance of EGFR in brain tissue*

EGFR activity was detectable in only one of 11 samples of normal brain (9%), in 1/6 schwannomas (16%), 20/21 samples of meningioma (95%, median level 155 fmol mg$^{-1}$ protein), in 2/3 metastatic deposits from extracranial malignant tumours and in 41/55 intracranial tumours (75%, median level 44 fmol mg$^{-1}$ protein). Within the gliomata, there were marked differences between the levels of EGFR activity in the different histopathological types of tumour (Table IV), with very high levels being detected in some samples of glioblastoma multiforme.

By univariate analysis, the patient's age, tumour type and mode of primary treatment were significantly related to survival (Table V) whilst sex, type of specimen (1° or recurrence) assayed and EGFR concentration had no influence. Patients who were younger at presentation fared better than those presenting later and survival related to tumour histological type in the manner expected, i.e. increasing survival in the order (1) glioblastoma multiforme, (2) anaplastic astrocytoma, (3) oligodendroglioma/mixed oligo-astrocytoma, (4) PNET and (5) astrocytoma. In addition, patients treated by surgery alone fared less well than those receiving combined treatments.

Even though patients with high levels of EGFR activity appeared to fare worse than those with lower levels (e.g.

**Figure 3** The time course of binding at three different temperatures in three types of tissue: **a**, rat liver, **b**, breast tumour and **c**, brain tumour.

**Table II** Relationship of various factors to prognosis in patients with breast cancer by univariate analysis

| | *Significance level (P)* | |
|---|---|---|
| *Factor* | *DFI* | *Survival* |
| 'Node status'[a] | 0.0004 | 0.018 |
| Tumour size | 0.014 | 0.15 |
| ER concentration | 0.018 | 0.029 |
| Tumour stage | 0.079 | 0.048 |
| Assay specimen[b] | 0.24 | 0.79 |
| EGFR concentration | 0.27 | 0.45 |
| Age | 0.68 | 0.57 |
| Menstrual status | 0.70 | 0.73 |
| Treatment | 0.71 | 0.53 |

[a]'Node status' = pathological node status where known, ($n$ = 84) plus 'clinical node status' where former unknown ($n$ = 42); [b]Assay specimen refers to whether the assay was conducted on primary or secondary tumour.

**Table III** Relationship of various factors to prognosis in patients with breast cancer by multivariate analysis

| | *Significance level (P)* | |
|---|---|---|
| *Factor* | *DFI* | *Survival* |
| *Factors included in the model:* | | |
| 'Node status'[a] | 0.0004 | 0.018 |
| ER concentration | 0.0018 | 0.011 |
| *Factors rejected from the model:* | | |
| Tumour stage | 0.12 | 0.61 |
| Tumour size | 0.12 | 0.86 |
| Menstrual status | 0.34 | 0.39 |
| Treatment | 0.42 | 0.67 |
| EGFR concentration | 0.60 | 0.99 |
| Assay specimen[b] | 0.66 | 0.22 |
| Age | 0.81 | 0.75 |

[a]'Node status' = pathological node status where known ($n$ = 84) plus 'clinical node status' where former unknown ($n$ = 42); [b]Assay specimen refers to whether the assay was conducted on primary or secondary tumour.

**Table IV** Incidence and levels of EGFR activity in 96 intra-cranial tissues

| *Tissue* | *No* | *No[a] EGFR+ (%)* | *Median level of EGFR activity (fmol mg$^{-1}$ protein)* |
|---|---|---|---|
| Normal brain | 11 | 1 (9%) | 0 |
| Schwannoma | 6 | 1 (16%) | 0 |
| Meningioma | 21 | 20 (95%) | 155 |
| Glioblastoma multiforme | 28 | 26 (93%) | 214 |
| Anaplastic astrocytoma | 5 | 4 (80%) | 28 |
| Astrocytomas | 7 | 2 (29%) | 0 |
| Oligodendrogliomas /mixed oligo-astrocytomas | 7 | 5 (71%) | 50 |
| PNET | 4 | 2 (50%) | 2 |
| Rarer[b] malignancies | 4 | 2 (50%) | 22 |
| Metastatic deposits | 3 | 2 (66%) | 5 |

PNET = primitive neuroectodermal tumour. [a]Tissues showing any evidence of specific binding (±) and clear positives (+) were taken as EGFR+ for this calculation; [b]Two cerebellar haemangioblastomas, one sarcoma, one gliosarcoma.

**Table V** Relationship of various factors to survival in 51 patients with primary cerebral neoplasms by univariate analysis

| *Factor* | *Significance level (P)* |
|---|---|
| Age | <0.0001 |
| Tumour type | 0.0001 |
| Treatment | 0.029 |
| Sex | 0.064 |
| EGFR concentration | 0.14 |
| Assay specimen | 0.29 |

Assay specimen refers to whether assay was conducted on primary or secondary tumour.

Figure 4a), this effect was due to the association between high levels of EGFR activity and a particualr tumour type (glioblastoma multiforme) and the apparent influence of EGFR level on survival disappeared when only a single tumour type was considered (Figure 4b). By multivariate analysis, again only age, tumour type and mode of treatment influenced survival (Table VI), other factors being rejected from the model.

### Discussion

In this paper, we have established and examined an assay for EGFR. For this assay, we have preferred to separate free

**Figure 4** The relationship between EGFR activity and survival in **a**, all patients with intra-cranial tumours. **b**, shows the relationship in patients with glioblastoma multiforme. The number of patients at risk of death at 0, 9 and 18 months were respectively.

**a** all patients

EGFR − 12, 8 and 4

EGFR + (1–99 fmol mg$^{-1}$ protein) 19, 8 and 5

EGFR ++ (100–1000 fmol mg$^{-1}$ protein) 10, 6 and 2

EGFR +++(>1000 fmol mg$^{-1}$ protein) 10, 3 and 1

**b** patients with glioblastoma multiforme

EGFR − 2, 1 and 0

EGFR + (1–99 fmol mg$^{-1}$ protein) 9, 4 and 1

EGFR ++ (100–1000 fmol mg$^{-1}$ protein) 7, 3 and 0

EGFR +++(>1000 fmol mg$^{-1}$ protein) 10, 3 and 1

**Table VI** Relationship of various factors to survival in 51 patients with primary cerebral neoplasms: multivariate analysis

| *Factor* | *Probability value (P)* |
|---|---|
| *Factors included in the model* | |
| Treatment | 0.0019 |
| Tumour type | 0.0026 |
| Age | 0.021 |
| *Factors rejected from the model* | |
| Sex | 0.22 |
| Assay specimen | 0.61 |
| EGFR concentration | 0.65 |

Assay specimen refers to whether assay was conducted on primary or secondary tumour.

and bound hormone by co-precipitation, and to measure the total receptor site concentration present in the total particulate fraction of the cell. Others have used a variety of ligand-binding methods (e.g. Nicholson *et al.*, 1988), or an autophosphorylation method (Lieberman *et al.*, 1985), and slightly different subcellular fractions (e.g. Sainsbury *et al.*, 1985; Fitzpatrick *et al.*, 1984; Perez *et al.*, 1984). Most tissues, in our experience, demonstrated both high and low affinity binding sites; since the significance of these two types of site is unclear, though it might relate to the degree of aggregation of the EGFR monomers (Schlessinger, 1988) and since it was technically simpler, we preferred to measure total sites. Equally, since EGFR binding activity was found in all the subfractions of the cell, for ease of preparation and for maximal sensitivity, we assayed the total receptor sites present in the cell. Of the compounds tested, only EGF of human or mouse origin significantly displaced $^{125}$I EGF from binding in three types of tissue examined; on a molar basis, we did not observe any consistent difference in relative binding affinity between EGFs from the two different sources.

Despite the above examination of the assay method, while the assay of samples of placenta and rat liver was straightforward, some difficulties were encountered with samples from tumours of brain or breast. For the former, binding was occasionally very high or gave anomalous Scatchard plots with apparently low affinity binding ($K_d$ 10–60 nM) even when reassayed at lower dilutions of the membranes. This coupled with the different time course of binding suggested that the EGFR in brain tumours may differ from those in other tissues, or that the apparent differences were due to technical factors (tissue damage at/after operation, or assay of samples containing too many receptors for saturation). For the breast tumours, in general, the binding of EGF was very low and thus accurate separation of the Scatchard plot into two components would have been difficult. These analyses of EGFR activity, therefore, are probably quantitatively approximate, but qualitatively accurate.

EGF receptor activity was detected in 11% (1/9) samples of benign breast tissue and 9% (1/11) samples of normal brain and in agreement with the immunocytochemical findings of Horsfall *et al.* (1989), in virtually all the meningiomas (20/21 = 95%) examined. The significance of the latter observation is as yet unclear, though like breast cancers, with which they show an association (Rubinstein & Schein, 1989), meningiomas also may contain steroid receptors (Whittle *et al.*, 1987; Horsfall *et al.*, 1989). Maligant tumours of the breast contained some binding activity in 43% of the 131 tumours examined, whilst those of the brain contained activity in 75% of the 55 intracranial cases.

In the case of breast cancer, our study population was a mixture of patients at all stages in the course of the disease, treated at nine different hospitals. They represent a group of patients with larger tumours and a poor prognosis; there were 43 recurrences and 30 deaths in the relatively short follow-up period, and only 63% of the tumours contained detectable ER activity (c.f. ≥75% in unselected patients in our experience – Hawkins *et al.*, 1987). By both univariate and multivariate analyses, in contrast to the findings of the Newcastle group (Sainsbury *et al.*, 1985, 1987; Nicholson *et al.*, 1989), EGFR measurements were of no prognostic value and only node status and ER measurements related significantly to disease-free interval or survival (Table III). However, it is to be noted that EGFR and ER show a strong inverse relationship, and thus to some extent, they provide the same information. The reasons for the difference between our findings and the previous reports are not clear but they could relate to (1) insufficient sensitivity in our EGFR assay, particularly where only small samples of tumour were available, (2) our study of total EGFR sites (c.f. high affinity sites by the Newcastle workers), though this seems unlikely since EGFR + tissues generally contained high affinity sites and (3) the fact that the tumours studied constitute a selected group which is probably not representative of the spectrum of breast cancers. Other workers have also examined the prognostic value of EGFR measurements but the results have been contradictory. Grimaux *et al.* (1989), examining a small subgroup of 55 patients, found that EGFR status was an important prognostic factor by both univariate and multivariate analyses at 40 months of follow-up. By contrast, Foekens *et al.* (1989), in a larger group of 214 patients, found that, as in the present study, steroid receptor status, but not EGFR status, was of prognostic value.

For brain tumours, to our knowledge, the prognostic value of EGFR measurements has not been examined previously. In the present series, although there was a tendancy for tumours of the most aggressive histological type (glioblastoma multiforme) to produce high levels of EGFR, multivariate analysis revealed that only age, histopathological tumour type and type of treatment related to survival.

Our findings of high levels of expression of EGFR by some gliomata are in agreement with those of others (Liebermann *et al.*, 1985; Wong *et al.*, 1987; Helseth *et al.*, 1988). This overexpression has been found frequently to be associated with gene amplification (Liebermann *et al.*, 1985; Helseth *et al.*, 1988) and the process appeared to confer a growth advantage upon suspensions of single cells from human gliomata, grown in agar (Helseth *et al.*, 1988). Glioma cell lines in culture are, in some cases, sensitive to EGF and can be inhibited by an antibody against the EGFR (Werner *et al.*, 1988). *In vivo*, four of seven patients with recurrent gliomata localised by immunoscintigraphy using $^{125}$I-labelled antibodies against EGFR and treated with $^{131}$I-labelled antibody have shown clinical improvement (Kalofonos *et al.*, 1989). Despite these observations, Collins (Collins, P. Sweden - personal communication to I.R.W.), in line with our own findings on EGFR expression, found no correlation between EGFR gene amplification and survival.

In summary, we conclude that the EGFR protein tends to be expressed in malignant tumours of poorer prognosis in both breast and brain. However, the extent of this expression in variable and not sufficiently consistent, by our mode of analysis, to be of prognostic significance. Re-examination of this question with a more sophisticated and sensitive assay or in a wider spectrum of breast cancers could alter that conclusion.

We wish to thank particularly, members of the Department of Surgery in Newcastle, Mr Richard Sainsbury, Mrs Pauline Chambers and Clive Hetherington (Biochemistry) for help and discussion of their techniques. Dr Tony Bramley, MRC Centre for Reproductive Biology, Edinburgh also helped by discussion and provision of the details of his co-precipitation technique.

We thank Dr Jeanne Bell, Department of Neuropathology, University of Edinburgh for classifying the intra-cranial tumours and other members of the Department of Pathology for selecting the breast cancer tissue for assay and for confirmation/otherwise of the presence of tumour. Dr Ian Nimmo, Department of Biochemistry, University of Edinburgh also helped with advice and discussion of the computer analysis of Scatchard plots.

Dr H. Gregory, ICI Ltd, kindly provided us with the sample of recombinant human EGF and Dr D. Pepper, Scottish National Blood Transfusion Service Headquarters Laboratory, 2 Forrest Road, Edinburgh gave us a preparation enriched in PDGF.

We are grateful to Dr Helen J. Stewart, Director of the Scottish Cancer Trials Office (MRC), Professor D.C. Carter and Professor Sir Patrick Forrest for advice and encouragement. Ms Elizabeth Hunter and Ji Xian Wang, Medical Statistics Unit, kindly helped with the analysis of the data.

The work was supported, in part, by funding from the Melville Trust to Mr Whittle and Dr Hawkins.

Oestrogen receptor assays were performed by Ann L. Tesdale and David Carson with the support of the Lothian Health Board.

### References

ADAMI, H.O., GRAFFMAN, S., LINDGREN, A. & SALLSTROM, J. (1985). Prognostic implication of estrogen receptor content in breast cancer. *Breast Cancer Res. Treatment*, **5**, 293.

BATTAGLIA, F., SCAMBIA, G., ROSSI, S. & 8 others (1988). Epidermal growth factor receptor in human breast cancer: correlation with steroid hormone receptors and axillary lymph node involvement. *Eur. J. Cancer Clin. Oncol.*, **24**, 1685.

BRADFORD, M.M. (1976). A rapid and sensitive method for the quantitation of microgram quantities of protein utilizing the principle of protein-dye binding. *Analyt. Biochem.*, **72**, 248.

BURGER, P.C., VOGEL, F.S., GREEN, S.B. & STRIKE, T.A. (1985). Glioblastoma multiforme and anaplastic astrocytoma: pathological criteria and prognostic implications. *Cancer*, **56**, 1106.

CARPENTER, G. (1985). Binding assays for epidermal growth factor p101-110. In *Methods in Enzymology*, **109**, Academic Press.

COHEN, J. (1960). A coefficient of agreement for nominal scales. *Educational and Pyschological Measurement*, **20**, 37.

COWLEY, G.P., SMITH, J.A. & GUSTERSON, B.A. (1986). Increased EGF receptors on human squamous carcinoma cell lines. *Br. J. Cancer*, **53**, 223.

DELARUE, J.C., FRIEDMAN, S., MOURIESSE, H., MAY-LEVIN, F., SANCHO-GARNIER, H. & CONTESSO, G. (1988). Epidermal growth factor receptor in human breast cancers: correlation with estrogen and progesterone receptors. *Breast Cancer Res. Treatment*, **11**, 173.

EPENETOS, A.A., COURTENAY-LUCK, N., PICKERING, D. & 4 others (1985). Antibody-guided irradiation of brain glioma by arterial infusion of radioactive monoclonal antibody against epidermal growth factor receptor and blood group A antigen. *Br. Med. J.*, **290**, 1463.

FITZPATRICK, S., BRIGHTWELL, J., WITTLIFF, J.L., BARROWS, G.H. & SCHULTZ, G.S. (1984). Epidermal growth factor binding by breast tumour biopsies and relationship to estrogen and progestin receptor. *Cancer Res.*, **44**, 3448.

FOEKENS, J.A., PORTENGEN, H., VAN PUTTEN, W.L.J. & 4 others (1989). Prognostic value of receptors for insulin-like growth factor I, Somatostatin and epidermal growth factor in human breast cancer. *Cancer Res.*, **49**, 7002.

FRAKER, P.J. & SPECK, J.C. (1978). Protein and cell membrane iodination with a sparingly soluble chloramide 1,3,4,6 tetrachloro 3α6αdiphenylglyrouril. *Biochem. Biophys. Res. Commun.*, **80**, 849.

FRAPPAZ, D., SINGLETARY, S.E., SPITAZER, G. & YUNG, A. (1988). Enhancement of growth of primary metastatic fresh human tumours of the nervous system by epidermal growth factor in serum-free short term culture. *Neurosurgery*, **23**, 355.

GRIMAUX, M., ROMAINS, S., REMVIKOS, Y., MARTIN, P.M. & MAGDELENAT, H. (1989). Prognostic value of epidermal growth factor in node-positive breast cancer. *Breast Cancer Res. Treatment*, **4**, 77.

HAWKINS, R.A., BLACK, R., STEELE, R.J.C., DIXON, J.M.J. & FORREST, A.P.M. (1981). Oestrogen receptor concentration in primary breast cancer and axillary node metastases. *Breast Cancer Res. Treatment*, **1**, 245.

HAWKINS, R.A., HILL, A. & FREEDMAN, B. (1975). A simple method for the determination of oestrogen receptor concentrations in breast tumours and other tissues. *Clinica Chimica Acta*, **64**, 203.

HAWKINS, R.A., WHITE, G., BUNDRED, N.J. & 4 others (1987). Prognostic significance of oestrogen and progestogen receptor activities in breast cancer. *Br. J. Surgery*, **74**, 1009.

HELSETH, E., UNSGAARD, G., DALEN, A. & 4 others (1988). Amplification of the epidermal growth factor receptor gene in biopsy specimens from human intracranial tumours. *Br. J. Neurosurgery*, **2**, 217.

HORSFALL, D.J., GOLDSMITH, K.G., RICCIARDELLI, C., SKINNER, J.M., TILLEY, W.D. & MARSHALL, V.R. (1989). Steroid hormone and epidermal growth factor receptors in meningiomas. *Aust. NZ Surg.*, **59**, 881.

HWANG, D.L., TAY, Y.C., LIN, S.S. & LEV-RAN, A. (1986). Expression of epidermal growth factor receptors in human lung tumours. *Cancer*, **58**, 2260.

KALOFONOS, H., PAWLIKOWSKA, T.R.B., SNOOK, D.E. & 4 others (1989). Radiolabelled monoclonal antibodies for localisation and therapy of brain gliomas. *Br. J. Cancer*, **59**, 318.

KNIGHT, W.A., LIVINGSTON, R.B., GREGORY, E.J. & MCGUIRE, W.L. (1977). Oestrogen receptor as an independent prognostic factor for early recurrence in breast cancer. *Cancer Res.*, **37**, 4669.

NEAL, D.E., MARSH, C., BENNETT, M.K. & 4 others (1985). Epidermal growth factor receptors in human lung tumours. *Cancer*, **58**, 2260.

NICHOLSON, S., SAINSBURY, J.R.C., HALCROW, P., CHAMBERS, P., FARNDON, J.R. & HARRIS, A.L. (1989). Expression of epidermal growth factor receptors associated with lack of response to endocrine therapy in recurrent breast cancer. *Lancet*, **i**, 182.

NICHOLSON, S., SAINSBURY, J.R.C., NEEDHAM, G.K., CHAMBERS, P., FARNDON, J.R. & HARRIS, A.L. (1988). Quantitative assays of epidermal growth factor receptor in human breast cancer: cut-off points of clinical relevance. *Int. J. Cancer*, **42**, 36.

PEREZ, R., PASCUAL, M., MACIAS, A. & LAGE, A. (1984). Epidermal growth factor receptors in human breast cancer. *Breast Cancer Res. Treatment*, **4**, 189.

PONDER, B. (1988). Gene losses in human tumours. *Nature*, **335**, 400.

RUBENSTEIN, A.B. & SCHEIN, M. (1989). The association of carcinoma of the breast with meningioma. *Surgery Gynecology & Obstetrics*, **169**, 334.

RUSSELL, D.S. & RUBENSTEIN, L.J. (1989). *Pathology of Tumours of the Nervous System*. E. Arnold: London, 5th edition.

SAINSBURY, J.R.C., FARNDON, J.R., NEEDHAM, G.K., MALCOM, A.J. & HARRIS, A.L. (1987). Epidermal growth factor receptor status as a predictor of early recurrence of and death from breast cancer: correlation with steroid hormone receptors and axillary lymph node involvement. *Eur. J. Cancer Clin. Oncol.*, **24**, 1685.

SAINSBURY, J.R.C., FARNDON, J.R., SHERBET, G.V. & HARRIS, A.L. (1985). Epidermal growth factor receptors and oestrogen receptors in human breast cancer. *Lancet*, **i**, 364.

SCATCHARD, G. (1949). The attraction of proteins for small molecules and ions. *Ann. NY Acad. Sci.*, **51**, 660.

SCHLESSINGER, J. (1988). Signal transduction by allosteric receptor oligomerisation. *TIBS*, **x**, 443.

SIMPSON, D.A. (1957). The recurrence of intracranial meningiomas after surgical treatment. *J. Neurol. Neurosurg. Psychiat.*, **20**, 22.

STEELE, R.J.C., HAWKINS, R.A., ANDERSON, T.J. & FORREST, A.P.M. (1987). The relevance of control histology in oestrogen receptor estimation. *Br. J. Cancer*, **56**, 517.

THORELL, J.I. & JOHNSON, B.G. (1971). Enzymatic iodination of polypeptides with $^{125}I$ to high specific activity. *Biochim. Biophys. Acta*, **251**, 363.

WERNER, M.H., HUMPHREY, P.A., BIGNER, D.D. & BIGNER, S.H. (1988). Growth effects of epidermal growth factor (EGF) and a monoclonal antibody against the EGF receptor on four glioma cell lines. *Acta Neuropathol. (Berlin)*, **77**, 196.

WHITTLE, I.R., HAWKINS, R.A. & MILLER, J.D. (1987). Sex hormone receptors in intra-cranial tumours and normal brain. *Eur. J. Surg. Oncol.*, **13**, 303.

WONG, A.J., BIGNER, S.H., BIGNER, D.D., KINZLER, K.W., HAMILTON, S.R. & VOGELSTEIN, B. (1987). Increased expression of the epidermal growth factor receptor gene in malignant gliomas is invariably associated with gene amplification. *Proc. Natl Acad. Sci. USA*, **84**, 6899.


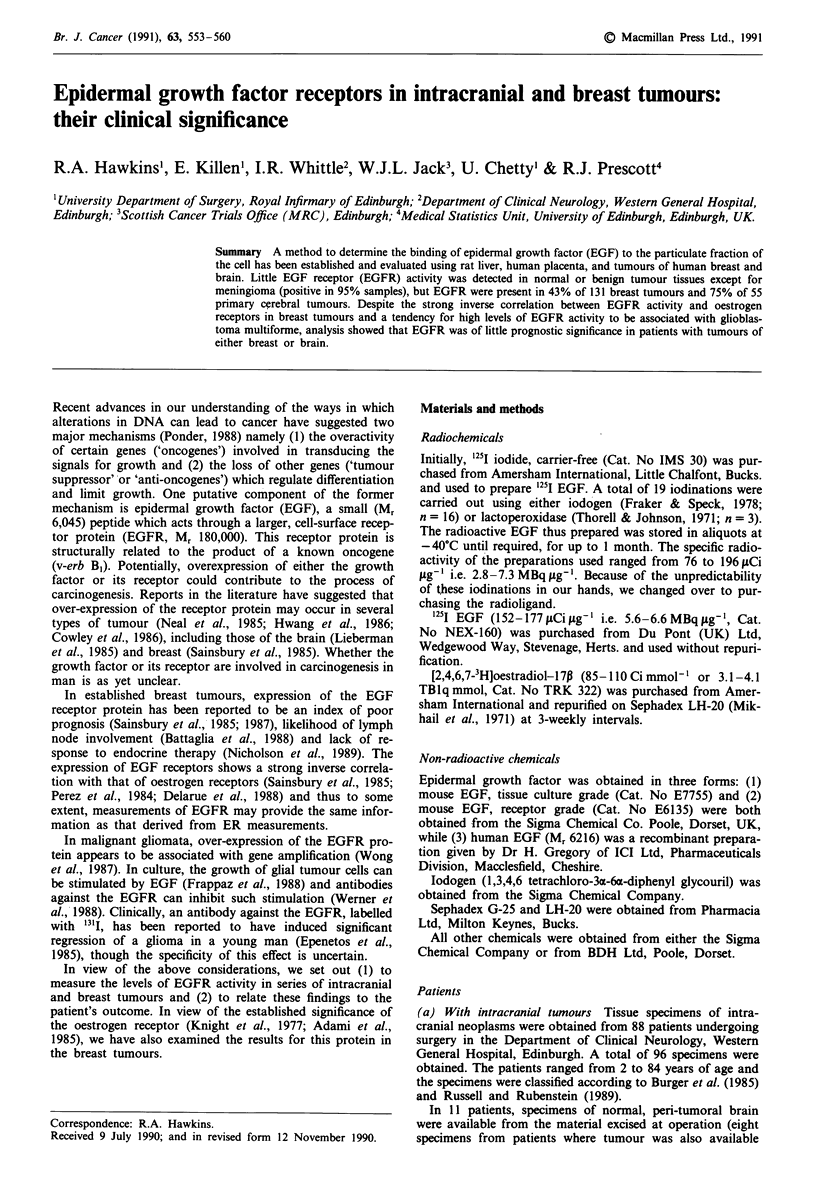

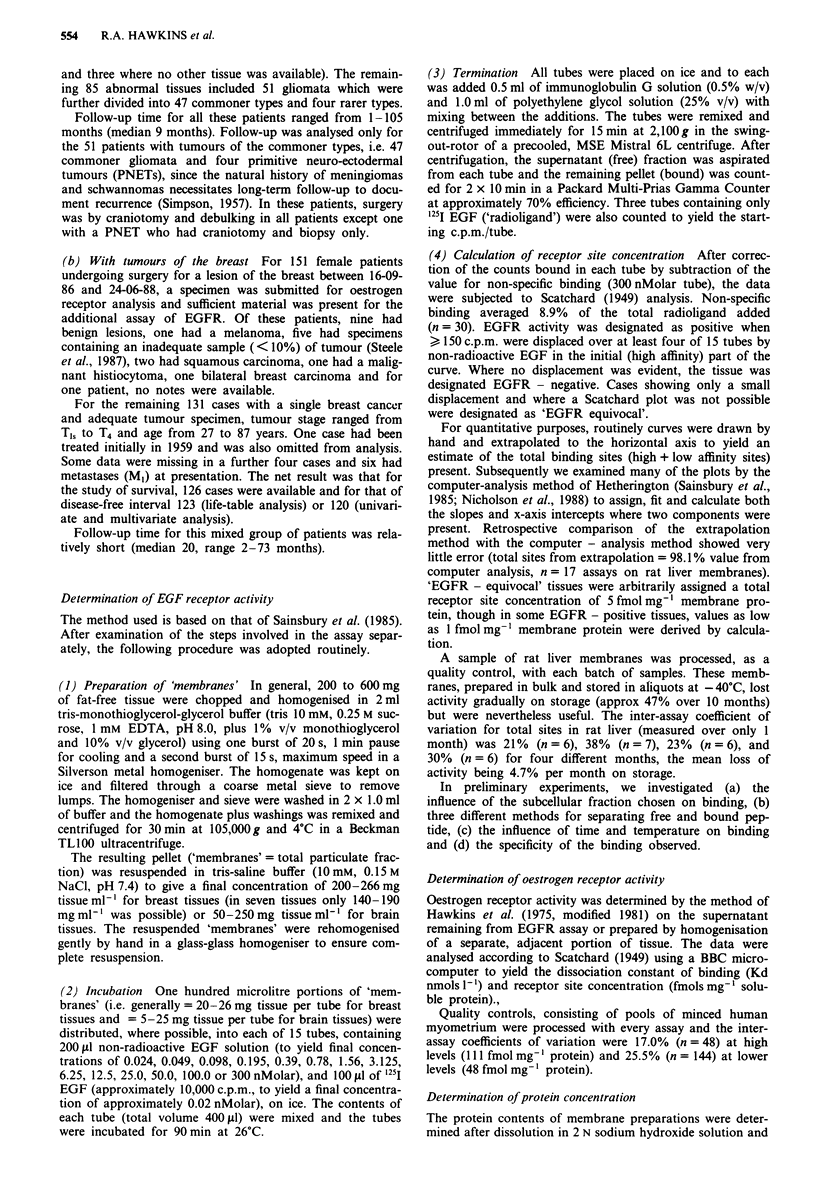

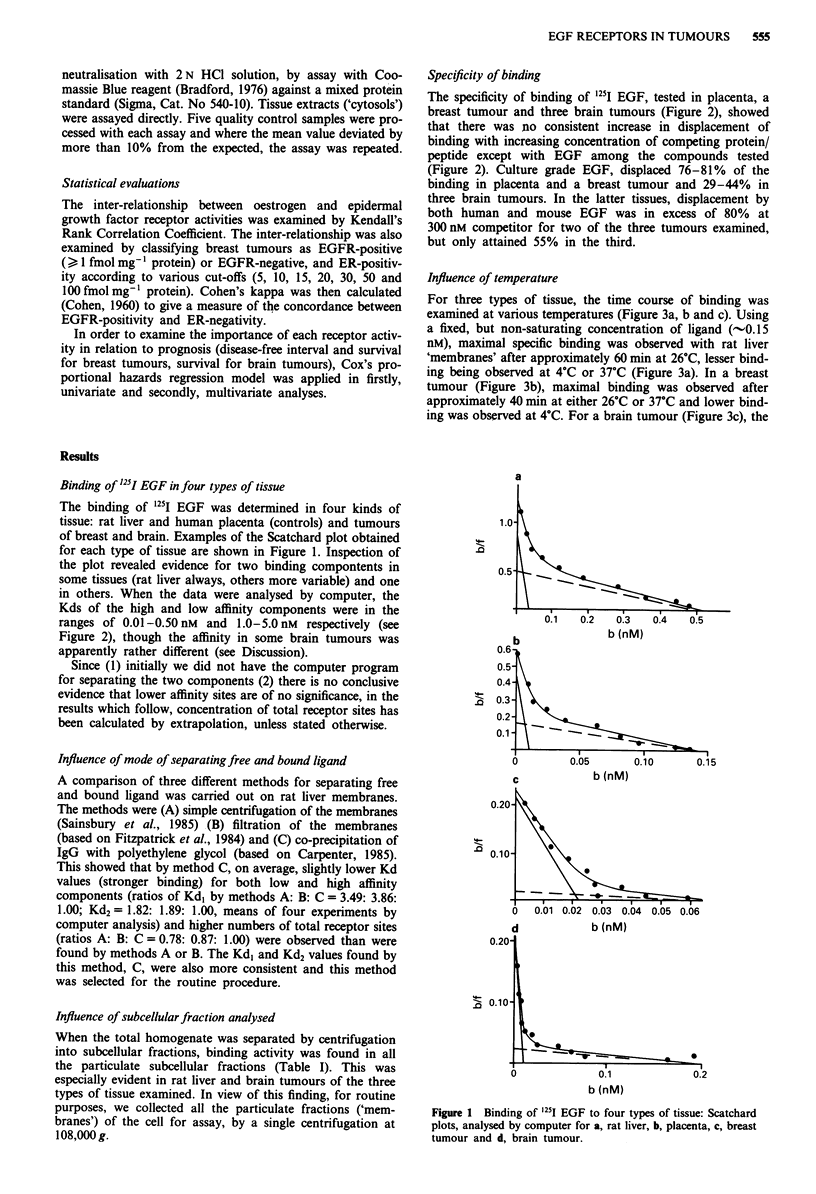

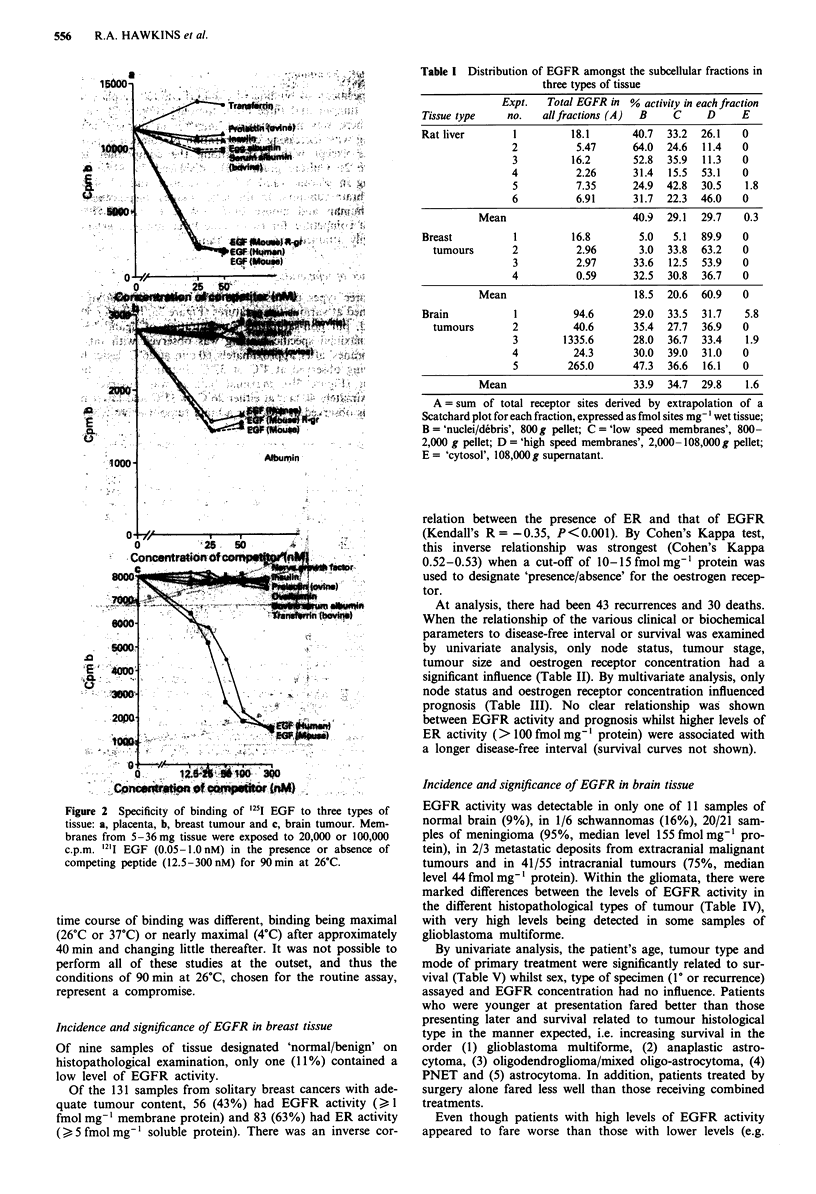

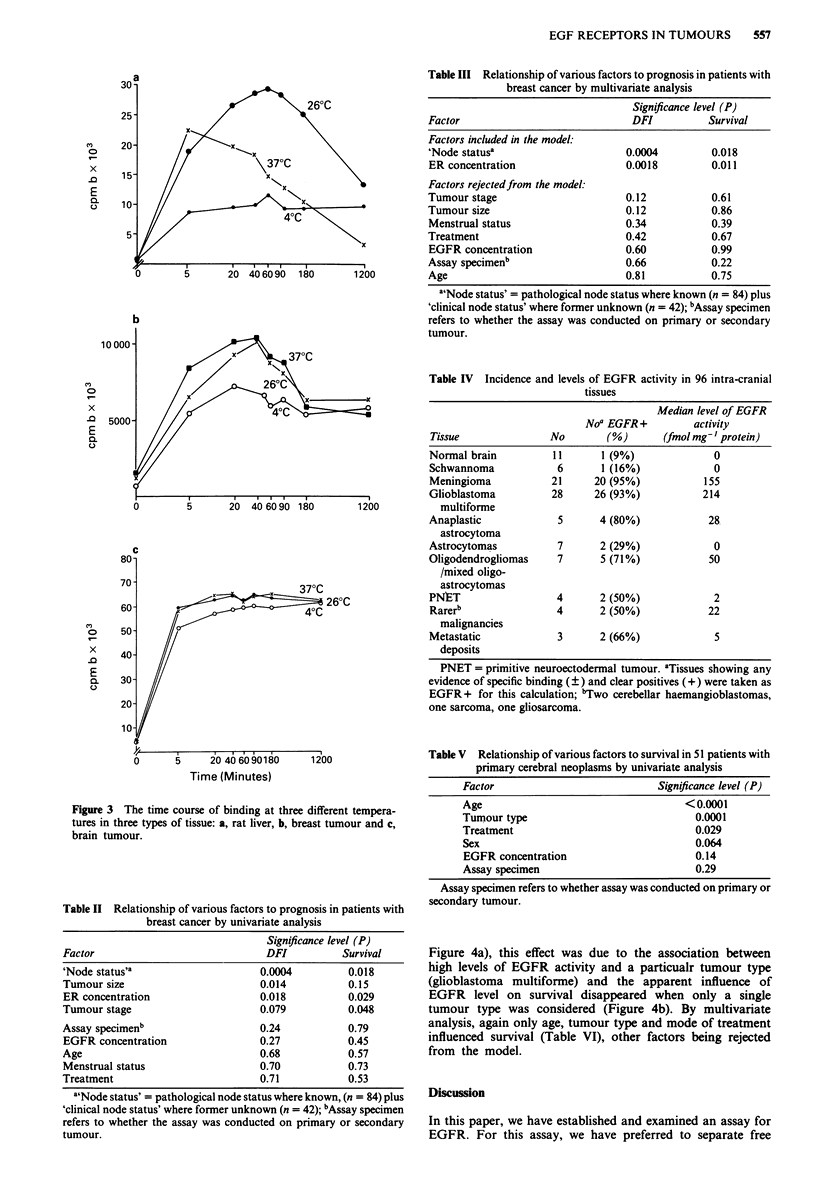

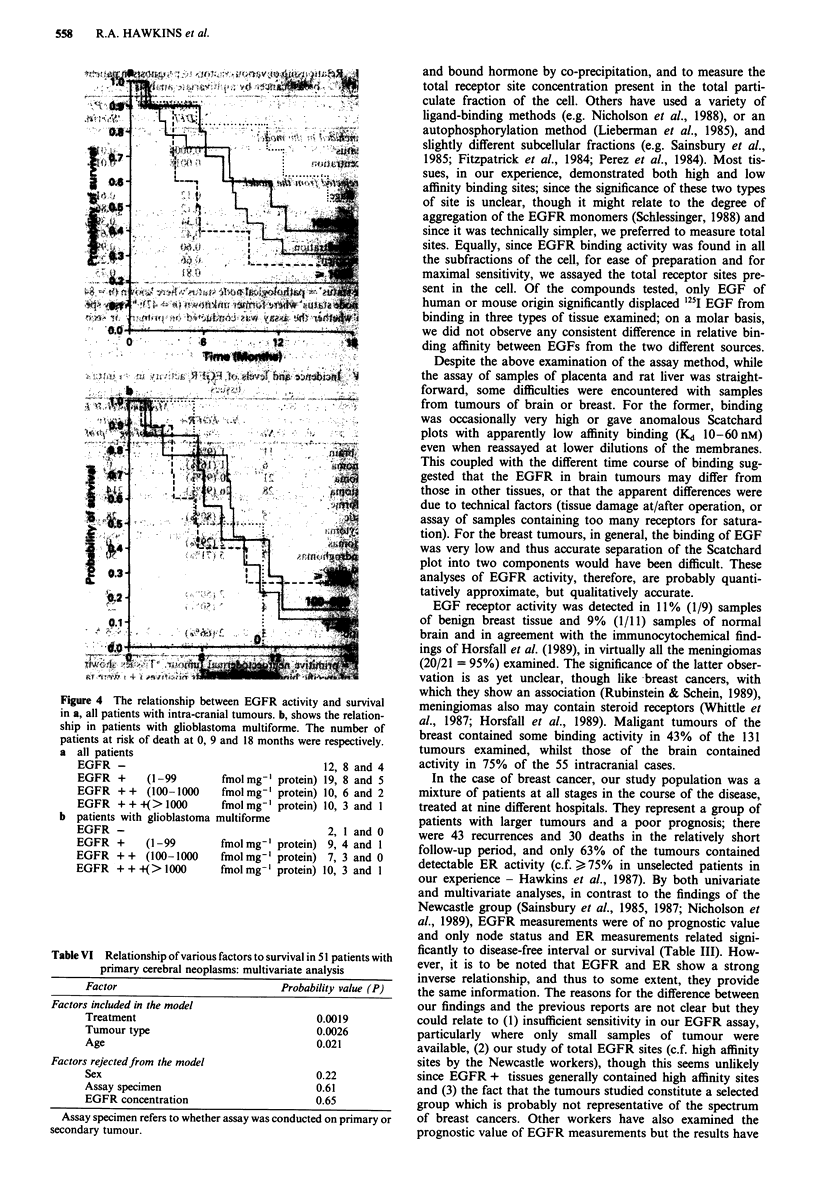

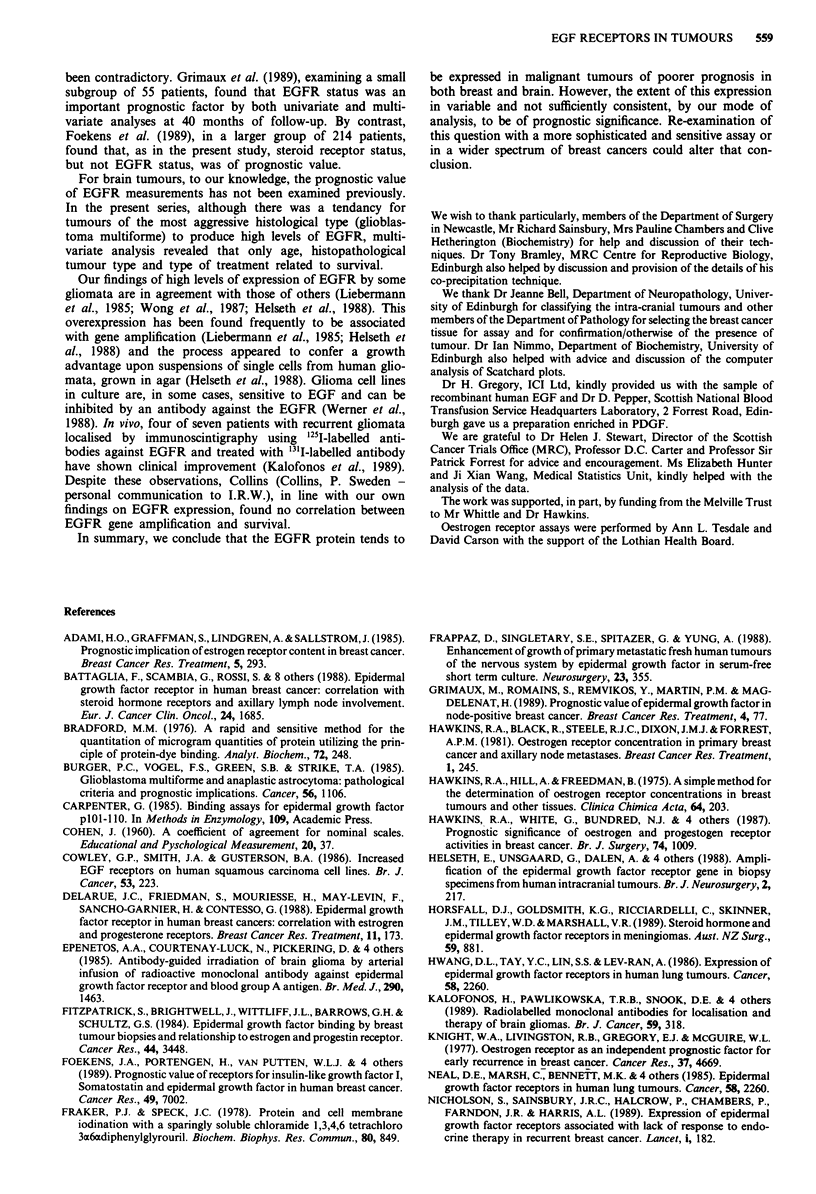

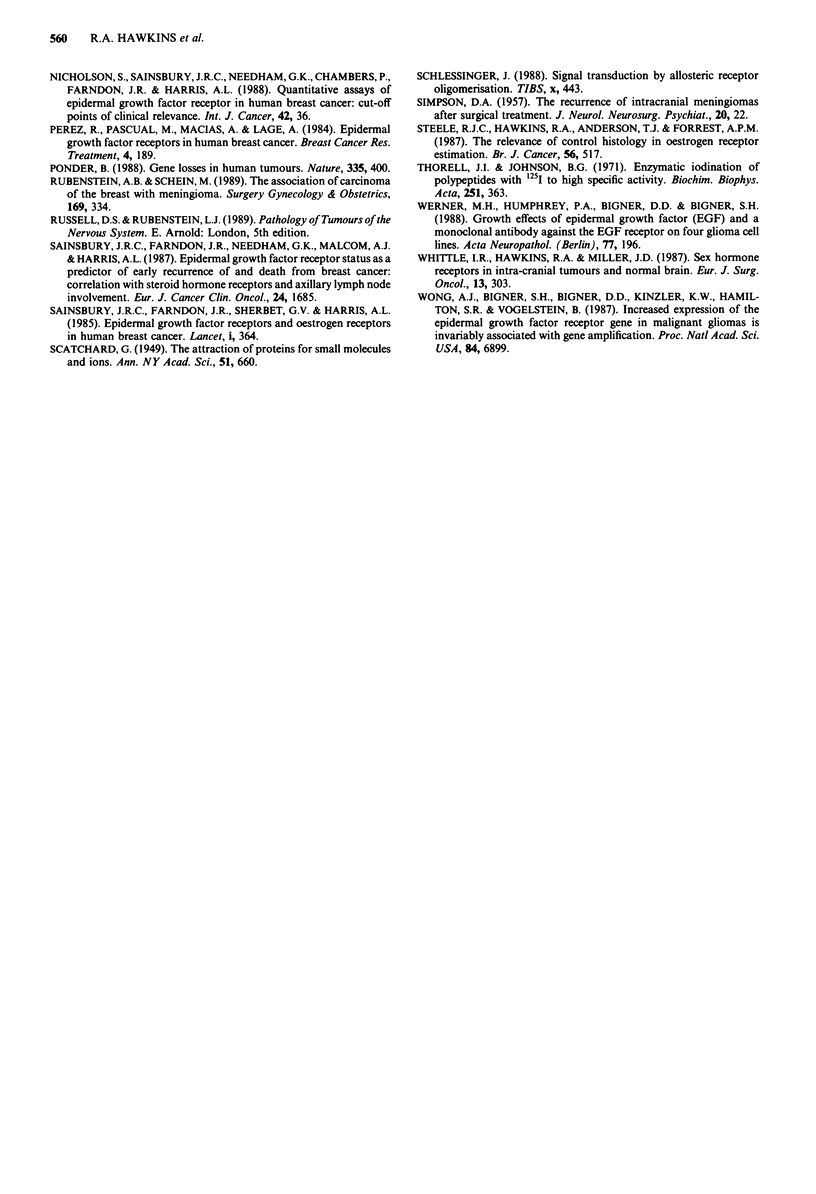


## References

[OCR_01019] Adami H. O., Graffman S., Lindgren A., Sällström J. (1985). Prognostic implication of estrogen receptor content in breast cancer.. Breast Cancer Res Treat.

[OCR_01024] Battaglia F., Scambia G., Rossi S., Panici P. B., Bellantone R., Polizzi G., Querzoli P., Negrini R., Iacobelli S., Crucitti F. (1988). Epidermal growth factor receptor in human breast cancer: correlation with steroid hormone receptors and axillary lymph node involvement.. Eur J Cancer Clin Oncol.

[OCR_01030] Bradford M. M. (1976). A rapid and sensitive method for the quantitation of microgram quantities of protein utilizing the principle of protein-dye binding.. Anal Biochem.

[OCR_01035] Burger P. C., Vogel F. S., Green S. B., Strike T. A. (1985). Glioblastoma multiforme and anaplastic astrocytoma. Pathologic criteria and prognostic implications.. Cancer.

[OCR_01048] Cowley G. P., Smith J. A., Gusterson B. A. (1986). Increased EGF receptors on human squamous carcinoma cell lines.. Br J Cancer.

[OCR_01053] Delarue J. C., Friedman S., Mouriesse H., May-Levin F., Sancho-Garnier H., Contesso G. (1988). Epidermal growth factor receptor in human breast cancers: correlation with estrogen and progesterone receptors.. Breast Cancer Res Treat.

[OCR_01058] Epenetos A. A., Courtenay-Luck N., Pickering D., Hooker G., Durbin H., Lavender J. P., McKenzie C. G. (1985). Antibody guided irradiation of brain glioma by arterial infusion of radioactive monoclonal antibody against epidermal growth factor receptor and blood group A antigen.. Br Med J (Clin Res Ed).

[OCR_01065] Fitzpatrick S. L., Brightwell J., Wittliff J. L., Barrows G. H., Schultz G. S. (1984). Epidermal growth factor binding by breast tumor biopsies and relationship to estrogen receptor and progestin receptor levels.. Cancer Res.

[OCR_01071] Foekens J. A., Portengen H., van Putten W. L., Trapman A. M., Reubi J. C., Alexieva-Figusch J., Klijn J. G. (1989). Prognostic value of receptors for insulin-like growth factor 1, somatostatin, and epidermal growth factor in human breast cancer.. Cancer Res.

[OCR_01077] Fraker P. J., Speck J. C. (1978). Protein and cell membrane iodinations with a sparingly soluble chloroamide, 1,3,4,6-tetrachloro-3a,6a-diphrenylglycoluril.. Biochem Biophys Res Commun.

[OCR_01082] Frappaz D., Singletary S. E., Spitzer G., Yung A. (1988). Enhancement of growth of primary metastatic fresh human tumors of the nervous system by epidermal growth factor in serum-free short term culture.. Neurosurgery.

[OCR_01093] Hawkins R. A., Black R., Steele R. J., Dixon J. M., Forrest A. P. (1981). Oestrogen receptor concentration in primary breast cancer and axillary node metastases.. Breast Cancer Res Treat.

[OCR_01099] Hawkins R. A., Hill A., Freedman B. (1975). A simple method for the determination of oestrogen receptor concentrations in breast tumours and other tissues.. Clin Chim Acta.

[OCR_01104] Hawkins R. A., White G., Bundred N. J., Dixon J. M., Miller W. R., Stewart H. J., Forrest A. P. (1987). Prognostic significance of oestrogen and progestogen receptor activities in breast cancer.. Br J Surg.

[OCR_01109] Helseth E., Unsgaard G., Dalen A., Fure H., Skandsen T., Odegaard A., Vik R. (1988). Amplification of the epidermal growth factor receptor gene in biopsy specimens from human intracranial tumours.. Br J Neurosurg.

[OCR_01115] Horsfall D. J., Goldsmith K. G., Ricciardelli C., Skinner J. M., Tilley W. D., Marshall V. R. (1989). Steroid hormone and epidermal growth factor receptors in meningiomas.. Aust N Z J Surg.

[OCR_01121] Hwang D. L., Tay Y. C., Lin S. S., Lev-Ran A. (1986). Expression of epidermal growth factor receptors in human lung tumors.. Cancer.

[OCR_01131] Knight W. A., Livingston R. B., Gregory E. J., McGuire W. L. (1977). Estrogen receptor as an independent prognostic factor for early recurrence in breast cancer.. Cancer Res.

[OCR_01139] Nicholson S., Sainsbury J. R., Halcrow P., Chambers P., Farndon J. R., Harris A. L. (1989). Expression of epidermal growth factor receptors associated with lack of response to endocrine therapy in recurrent breast cancer.. Lancet.

[OCR_01147] Nicholson S., Sainsbury J. R., Needham G. K., Chambers P., Farndon J. R., Harris A. L. (1988). Quantitative assays of epidermal growth factor receptor in human breast cancer: cut-off points of clinical relevance.. Int J Cancer.

[OCR_01158] Ponder B. (1988). Cancer. Gene losses in human tumours.. Nature.

[OCR_01153] Pérez R., Pascual M., Macías A., Lage A. (1984). Epidermal growth factor receptors in human breast cancer.. Breast Cancer Res Treat.

[OCR_01188] SIMPSON D. (1957). The recurrence of intracranial meningiomas after surgical treatment.. J Neurol Neurosurg Psychiatry.

[OCR_01175] Sainsbury J. R., Farndon J. R., Sherbet G. V., Harris A. L. (1985). Epidermal-growth-factor receptors and oestrogen receptors in human breast cancer.. Lancet.

[OCR_01184] Schlessinger J. (1988). Signal transduction by allosteric receptor oligomerization.. Trends Biochem Sci.

[OCR_01197] Thorell J. I., Johansson B. G. (1971). Enzymatic iodination of polypeptides with 125I to high specific activity.. Biochim Biophys Acta.

[OCR_01202] Werner M. H., Humphrey P. A., Bigner D. D., Bigner S. H. (1988). Growth effects of epidermal growth factor (EGF) and a monoclonal antibody against the EGF receptor on four glioma cell lines.. Acta Neuropathol.

[OCR_01208] Whittle I. R., Hawkins R. A., Miller J. D. (1987). Sex hormone receptors in intracranial tumours and normal brain.. Eur J Surg Oncol.

[OCR_01215] Wong A. J., Bigner S. H., Bigner D. D., Kinzler K. W., Hamilton S. R., Vogelstein B. (1987). Increased expression of the epidermal growth factor receptor gene in malignant gliomas is invariably associated with gene amplification.. Proc Natl Acad Sci U S A.

